# [Retracted] MicroRNA‑138 suppresses proliferation, invasion and glycolysis in malignant melanoma cells by targeting HIF‑1α

**DOI:** 10.3892/etm.2023.12353

**Published:** 2023-12-18

**Authors:** Yao Chen, Ke Cao, Shaohua Wang, Jia Chen, Bin He, Gu He, Yong Chen, Bin Peng, Jianda Zhou

Exp Ther Med 11:2513–2518, 2016; DOI: 10.3892/etm.2016.3220

Following the publication of this paper, it was drawn to the Editor’s attention by a concerned reader that the western blotting data in Fig. 2C and the Transwell assay data shown in Fig 3 were strikingly similar to data appearing in different form in other articles written by different authors at different research institutes that had either already been published, or were submitted for publication at around the same time.

Owing to the fact that the contentious data in the above article had already been published prior to its submission to *Experimental and Therapeutic Medicine,* the Editor has decided that this paper should be retracted from the Journal. The authors were asked for an explanation to account for these concerns, but the Editorial Office did not receive a reply. The Editor apologizes to the readership for any inconvenience caused.

